# The Mystery of Exosomes in Gestational Diabetes Mellitus

**DOI:** 10.1155/2022/2169259

**Published:** 2022-06-08

**Authors:** Tong Chen, Dan Liu

**Affiliations:** Department of Endocrinology and Metabolism, First Affiliated Hospital of Dalian Medical University, Dalian, Liaoning, China

## Abstract

Gestational diabetes mellitus (GDM) is one of the common pregnancy complications, which increases the risk of short-term and long-term adverse consequences in both the mother and offspring. However, the pathophysiological mechanism of GDM is still poorly understood. Inflammation, insulin resistance and oxidative stress are considered critical factors in the occurrence and development of GDM. Although the lifestyle intervention and insulin are the primary treatment, adverse pregnancy outcomes still cannot be ignored. Exosomes have a specific function of carrying biological information, which can transmit information to target cells and play an essential role in intercellular communication. Their possible roles in normal pregnancy and GDM have been widely concerned. The possibility of exosomal cargos as biomarkers of GDM is proposed. This paper reviews the literature in recent years and discusses the role of exosomes in GDM and their possible mechanisms to provide some reference for the prediction, prevention, and treatment of GDM and improve the outcome of pregnancy.

## 1. Introduction

Gestational diabetes mellitus (GDM) is defined as impaired glucose tolerance that occurs for the first time during pregnancy and incidence of GDM in accordance with the increase in obesity and type 2 diabetes mellitus (T2DM) prevalence. GDM increases the incidence of perinatal complications such as macrosomia, cesarean section, preterm delivery, neonatal hypoglycemia, and hyperbilirubinemia and the maternal risk of T2DM and cardiovascular disease in the long term. The pathogenesis of GDM is complex and has not been fully clarified, which is believed to be related to insulin resistance, inflammation, oxidative stress, adipose tissue, and endothelial cell dysfunction [1, 2]. It is of great clinical value to further study the pathogenesis of GDM and put forward innovative prevention and treatment methods.

Extracellular vesicles (EVs) are nano-sized particles derived from the membrane, which play the role of intercellular communication. The exosome, also known as small extracellular vesicles (sEVs), is a subgroup concerned by many researchers [3, 4]. Exosomes are extracellular vesicles produced by various cells in the diameter of 50-150 nm, which are widely found in peripheral blood, urine, saliva, cerebrospinal fluid, and placenta. Exosomes carry cargos rich proteins, lipids, DNA, RNA, and other bioactive factors, which are released in the form of exocytosis to take a part in the process of intercellular information transmission, immune regulation, antigen presentation, tumor growth, and so on. At present, many studies have shown that exosomes are involved in the occurrence and development of a variety of diseases and may be used in the treatment of diseases. During pregnancy, all types of cells in the body secrete exosomes, including placenta, adipose tissue, liver, pancreas, and skeletal muscle [5]. Higher plasma concentrations of exosomes were observed in normal pregnant women than in nonpregnant women [6]. In addition, placental-derived exosomes were detected in maternal plasma as early as six weeks of gestation, and the concentration of exosomes gradually increased with gestation age [7]. The exosomes isolated from maternal blood have biological activity in vitro and can enter target cells through endocytosis [8]. Maternal sEVs can circulate from the mother to the fetus through the placental barrier, resulting in functional changes during pregnancy [9–11], indicating that sEVs play an important role in maintaining communication between the fetus and the mother [10]. Moreover, sEVs are also associated with GDM, a complication during pregnancy [12]. This article will introduce the possible effects of exosomes derived from different tissue and their possible mechanisms of influence on GDM.

## 2. Exosomes Derived from Different Tissue That Related with GDM

### 2.1. Placental-Derived Exosomes

As an important organ connecting mother and fetus, the placenta plays a crucial role in regulating maternal physiological function and nutritional exchange between mother and infant during pregnancy. Placental-derived exosomes are secreted by various placental cells during pregnancy, mainly syncytiotrophoblastic layer releasing a large amount of EVs through exocytosis [13, 14]. Placental-derived exosomes can be identified by human placental alkaline phosphatase (PLAP) because PLAP is a specific allosteric synthesized in the placenta [6, 15]. Compared with the control group, the particle size of exosomes in umbilical cord blood in the GDM group was larger and the concentration was higher [16]. It was well established that the concentration of placental-derived exosomes in patients with GDM was significantly higher than that in normal pregnant women [17], suggesting that maternal hyperglycemia can promote the release of placental exosomes into blood circulation. Furthermore, maternal hyperglycemia can also stimulate the biological activity of placental-derived exosomes [18]. Additionally, placental-derived EVs can regulate insulin sensitivity during normal pregnancy, while insulin signaling is weakened in placental-derived EVs of GDM [19].

### 2.2. Adipose Tissue-Derived Exosomes

Adipose tissue is mainly composed of adipocytes, whose function is to store fat. Exosomes have been isolated from the culture medium of adipose cells, adipose tissue, and adipose-derived stem cells [20, 21]. The amount of maternal fat increases during pregnancy, but adipose tissue hyperplasia and hypertrophy are associated with insulin resistance and abnormal metabolism [22]. It was reported that the adipose tissue of patients with GDM secreted more sEVs than normal pregnant women, and the increase in the number of sEVs was believed to be positively correlated with the *Z* score of birth weight of offspring [23], suggesting that a higher level of exosomes in adipose tissue may be involved in regulating fetal growth. However, this differs from Franzago et al. who found that the concentration of sEVs in adipocytes in the maternal circulation of GDM was lower than that in normal pregnancy [24]. Different conditions such as hypoxia and obesity can lead to changes in the composition of exosomes [25]. A total of 509 proteins were detected in exosomes derived from adipose tissue of obese and non-obese diabetics, of which 200 proteins were differentially expressed in exosomes of obese diabetes [25]. Obesity is a risk factor and potential mechanism of GDM [26]. Obesity during pregnancy can cause systemic inflammation. The increase of circulating proinflammatory cytokines in adipose tissue may result in the rise of placental inflammatory cytokines secreted by the placenta, thus changing the placental function, which may promote the occurrence of GDM [5]. Additionally, exosomes derived from adipose tissue in GDM are rich in proteins targeting key pathways, such as mitochondrial dysfunction, OXPHOS, and SIRT signaling pathways, and exosomes derived from adipose tissue of GDM have the ability to increase the expression of genes related to glycolysis and gluconeogenesis in placental cells [23], which also suggests that adipose tissue-derived sEVs may be associated with the pathogenesis of GDM.

## 3. The Possible Mechanism of Exosomes Participation in GDM

### 3.1. Regulation of Gene Expression through RNA

Exosomes contain many different types of RNA molecules, such as miRNA, circRNA, and lncRNA. These RNA molecules can act on receptor cells and exert biological functions such as intercellular signal transmission.

#### 3.1.1. miRNA

miRNA is a wealthy class of small noncoding RNAs, which play a role in many biological processes by specifically binding to its target mRNA to induce its degradation or inhibit translation. More and more studies have found that some miRNAs involved in placental and fetal development are abnormally regulated in GDM, suggesting that miRNAs may be involved in the pathogenesis of GDM [8, 27]. The chromosome 19 miRNA cluster (C19MC), as an exosomes carrier, is expressed only in the placenta [28], the most important of which are miR-516b-5p, miR-517-5p, and miR-518a-3p [29]. Placental-derived miRNAs are released from syncytiotrophoblast cells into the maternal circulation [30, 31], and their expression levels are regulated by stimuli such as stress, circulating blood glucose and other pregnancy characteristics. The abundance of miR-518a-5p, miR-518b, miR-518c, miR-518e, miR-520c-3p, and miR-525-5p in placental exosomes of GDM patients was higher than that of the normal pregnancy group, and miR-520c-3p was related to placental oxygen supply [18]. However, the cellular targets of C19MC miRNAs have not been fully determined, which may include non-trophoblastic placental cells, maternal organs or fetal cells.

In addition, some non-C19MC-coded miRNAs, such as miR-16-5p and miR-222-3p, are also related to GDM [32]. miR-16-5p plays a regulatory role in PI3K/Akt, Wnt, insulin, and mTOR signaling pathways, and the overexpression of these signaling pathways is related to GDM [33, 34]. The gene encoding insulin receptor substrate proteins 1 and 2 (IRS1/IRS2) are the targets of miR-16-5p. The upregulation of miR-16-5p in GDM patients at the 2nd trimester will lead to the negative regulation of IRS1 and IRS2, which may lead to abnormal Wnt/*β*-catenin signaling and eventually lead to diabetes [35, 36]. The expression of miR-222-3p in serum of GDM patients was lower than that of control group either at the 17th or 26th week of pregnancy [37, 38]. Nevertheless, Shi et al. proved that miR-222-3p was upregulated in the adipose tissue of GDM [39]. Most of the genes involved in the pathways of insulin resistance are the targets of miR-16-5p and miR-222-3p [14, 40]. miR-103 and miR-107 are also closely related to insulin sensitivity [41]. Other studies reported that the levels of miR-132, miR-29a, and miR-222 in serum of patients with GDM decreased [37], while the levels of hsa-miR-16-5p, hsa-miR-17-5p, hsa-miR-19a-3p, hsa-miR-19b-3p, and hsa-miR-20a-5p in plasma of patients with GDM are increased [32]. The expressions of miRNA-125b and miRNA-144 were out of balance in the circulating exosomes and placenta of patients with GDM [42]. In summary, exosome-derived miRNAs are becoming a new participant in the development of GDM, which may become an attractive biomarker and potential therapeutic target.

#### 3.1.2. circRNA

circRNA is a kind of endogenous noncoding RNA with wide distribution and diverse cellular functions. circRNAs can alter mRNA stability and inhibit protein activity by binding to RNA binding proteins [43]. circRNAs can also serve as templates for protein translation [44]. The enrichment and stability of circRNAs in exosomes have been discovered recently. circRNAs are involved in the development of GDM and fetal growth. A recent study shows that in patients with GDM, the expression of circ_0008285 was significantly upregulated, while that of circ_0001173 was decreased [45]. 88371 circRNAs in the umbilical cord blood exosomes of the two groups were evaluated, and the results showed that there was a differential expression of 507 circRNAs between GDM patients and control groups, of which 229 circRNAs were significantly upregulated in GDM patients and 278 circRNAs were downregulated considerably in GDM patients. Further circRNA/miRNA interaction analysis showed that most of the exosomal circRNAs contained miRNA binding sites and some of the miRNAs were related to GDM [46]. Exosomal circRNAs regulates gene expression through the miRNA sponge mechanism [47]. circRNAs have many miRNA binding sites that compete with miRNAs, so circRNAs may regulate gene expression by reducing the inhibition of miRNAs on target molecules. For example, miR-330, miR-23a, and miR-16-5p were upregulated in the plasma of patients with GDM [32, 48–50], and these miRNAs were paired with circ_0092108 downregulated in the exosomes of umbilical cord blood of patients with GDM. Therefore, the role of circRNAs in GDM may be related to the effect mediated by miRNAs. Nevertheless, the mechanism of circRNA-miRNA-target gene interaction needs to be further explored.

CircRNA polyribonucleotide nucleotidyltransferase 1 (Cir-PNPT1) is a newly discovered functional circRNA derived from the PNPT1 gene. It has been observed that it has different expression in GDM patients, which may be a risk factor for GDM [51]. Cir-PNPT1 was highly expressed in the placental tissues of GDM and high glucose (HG)-induced trophoblast cells. The expression of miR-889-3p decreased in GDM and HG-induced trophoblast cells, while the expression of PAK1 increased. Therefore, it is speculated that circ-PNPT1 may be involved in the inhibition of HG-induced trophoblast proliferation, migration, and invasion through the miR-889-3p/PAK1 axis [52]. Downregulation of circ-PNPT1 can alleviate trophoblast dysfunction induced by HG, suggesting that silencing circ-PNPT1 may play a protective role in the progression of GDM [52], which puts forward a new insight into the pathogenesis of GDM. In summary, exosomal circ-PNPT1 may be an ideal biomarker for GDM treatment.

#### 3.1.3. lncRNA

A study demonstrated that 84 mRNAs and 256 lncRNAs were differentially expressed in umbilical cord blood exosomes of patients with GDM compared with the control group. And further lncRNA/miRNA interaction analysis showed that most of the exosomal lncRNAs contained miRNA binding sites, some of which were related to GDM [16]. *β*-cell dysfunction is a pathophysiological characteristic of GDM. Some abnormal expressions of circulatory or placental-related lncRNAs are related to insulin resistance and *β*-cell dysfunction [53]. There is much evidence that exosomal lncRNAs can regulate miRNA-targeted gene expression, transcription, and protein synthesis [54, 55]. It has been reported that miR-362-5p and miR-508 were dysregulated in the placenta of patients with GDM [56]. miR-362-5p matched with lnc-ZNF800-1 : 1, which was confirmed to be downregulated in the umbilical cord blood exosomes of GDM, and miR-508 might combine with upregulated lnc-COX17-2 : 3. In consequence, exosomal lncRNAs may participate in GDM development and fetal growth through miRNA-mediated action. The potential mechanism of lncRNA-miRNA-target gene interaction in GDM is worthy of further studies. To sum up, exosomes play an essential role in GDM through RNA, especially miRNA. In Tables 1 and 2, we summarize the upregulated or downregulated expression levels of different types of RNA in GDM in recent years, all of which may be potential diagnostic and future targeted therapies for GDM.

### 3.2. Exosomes and Endothelial Cell Dysfunction

GDM mainly affects the function of placental vascular endothelial cells and then leads to the impairment of placental function. L-arginine/NO signaling pathway is one of the key signaling ways associated with vascular physiological changes and is associated with endothelial dysfunction. It was upregulated in GDM; that is, human cationic amino acid transporter 1 (hCAT-1) expression was increased, and eNOS activity and expression were increased [104–106]. Exosomes regulate the function of endothelial cells and are related to endothelial dysfunction [107, 108]. A few studies have shown that endothelial exosomes play a role as a regulator of L-arginine/NO signaling pathway, and endothelial exosomes participate in the regulation of fetal placental endothelial function in GDM by regulating PI3K/eNOS signaling pathway [109, 110]. A recent study has shown that exosomes from syncytiotrophoblast cells carry eNOS [111], which may lead to the production of NO. In addition, considering that exosomes in the fetal circulation of GDM may contain miRNA-203 that can induce NOS activity [112, 113], it may also affect the function of fetal endothelial cells. Exosomes released from human umbilical vein endothelial cells (HUVECs) were found to increase L-arginine transport through hCAT-1 [114, 115]. Interestingly, HUVECs from normal pregnant women blocked the increased L-arginine transport by GDM, the expression and activity of hCAT-1 and eNOS, and the activation of 44 and 42 kDa mitogen activated protein kinases (p44/42^mapk^). Inhibition of p44/42^mapk^ could reverse the increase of L-arginine uptake by GDM. This is helpful to understand the mechanism of foetoplacental endothelial dysfunction in GDM [115]. The above studies suggest that exosomes from patients with GDM may play a potential role in fetal circulation and induce foetoplacental endothelial dysfunction.

### 3.3. Exosomes and Inflammation

GDM is associated with inflammation [116]. Placental-derived EVs of GDM have biological activity, releasing a large amount of tumor necrosis factor *α* (TNF-*α*), granulocyte macrophage colony-stimulating factor (GM-CSF), interferon-*γ* (IFN-*γ*), IL,-6 and IL-8 [17]. In addition, others studies also demonstrated that HG can trigger the release of EVs by trophoblast cells and placental EVs has biological activity, which in turn increase the release of proinflammatory cytokines such as GM-CSF, IL-6, IL-8, IL-10, and TNF-*α* from endothelial cells [5, 18] and promote the proinflammatory state of pregnant women with GDM.

### 3.4. Exosomes and Oxidative Stress

GDM is characterized by hyperglycemia. Under the condition of hyperglycemia, the body produces more reactive oxygen species (ROS), which leads to oxidative stress. It is suggested that in patients with GDM, hyperglycemia and oxidative stress may induce exosomes production, affect its transport in the fetal-placental vascular system, and contribute to endothelial dysfunction and activation [115]. Exosomes affect oxidative stress. Exosomes from different tissues have been shown to induce target cells to produce ROS. Exposure of normal pregnant HUVECs to HUVECs exosomes of GDM can induce GDM phenotype and increase eNOS total protein and synthesis of NO [115]. On the other hand, exosomes of HUVECs in normal pregnancy can also reverse the functional abnormalities of these cells induced by HG [117]. It is worth noting to mention that clinical trials of common antioxidants in patients with GDM have shown that this method is ineffective in reducing oxidative stress [118, 119]. Exosomes from HUVECs from normal pregnancy and GDM did not affect the production of ROS in GDM HUVECs [115], while in a previous study, GDM increased ROS production in primary HUVECs culture [120]. In addition, some studies have suggested that the increase in exosomes release may be an adaptive response of cells to oxidative stress [121, 122].

### 3.5. Exosomes and Insulin Resistance

Insulin resistance is involved in the development of GDM. Adipose tissue plays a major role in the development of insulin resistance during pregnancy [123, 124], and adipose tissue-derived exosomes have been shown to affect biological processes such as metabolism, inflammation, regulation of glucose homeostasis, and insulin sensitivity [125]. A study [23] analyzed and compared the protein expression of exosomes from adipose tissue. It was found that the differentially expressed proteins in adipose tissue of GDM were related to mitochondrial dysfunction and targeting SIRT, OXPHOS, and EIF2 signaling pathways, which may be involved in the pathophysiological process of GDM; mTOR, eIF4, and p70S6K were also found. This suggested that this exosomal protein may be involved in the pathophysiological process of GDM by regulating the maternal environment [23]. The normal function of mitochondria is critical to cell metabolism because it controls ATP production and disposal of reactive oxygen species through OXPHOS. In addition, mitochondria have been shown to be associated with insulin resistance [126]. SIRT enhances mitochondrial metabolism and plays a synergistic role by regulating mitochondrial gene expression and post-translational modifications of mitochondrial enzymes [127, 128]. Another study reported that the downregulation of SIRT in adipose tissue was associated with decreased insulin sensitivity [129]. The activation of placental mTOR signal promotes mitochondrial function, protein synthesis, and the transport of nutrients such as amino acids, thus increasing the utilization of fetal nutrients. Compared with exosomes derived from normal glucose tolerance, proteins targeting the mTOR signaling pathway are enriched in exosomes derived from GDM. Therefore, exosomes derived from GDM may regulate placental nutritional capacity by activating placental mTOR signal [23]. In addition, it has been suggested that insulin resistance in pregnant women with GDM and their newborns may be due to decreased signals of protein kinase B/Akt (Akt) and mammalian target of rapamycin (mTOR) in human placental endothelial cells [130]. In addition, exosomes-derived placental of patients with GDM carry a specific set of miRNAs associated with skeletal muscle insulin signal transduction and insulin resistance [19].

Two proteins related to the regulation of insulin sensitivity were identified. It was found that the expression of PAPP-A was downregulated and the expression of CaMK2*β* was upregulated in exosomes of patients with GDM [131]. PAPP-A is a glycoprotein mainly synthesized by villous and extravillous cytotrophoblasts [132]. The concentration of PAPP-A in maternal circulation increases throughout pregnancy and decreases after birth. Low concentrations of PAPP-A in maternal circulation are associated with adverse pregnancy outcomes, including GDM [133]. It is worth noting that PAPP-A levels in the first trimester of pregnancy are associated with insulin resistance later in pregnancy [133, 134]. The low expression of PAPP-A in exosomes may play an important role in the intercellular communication between placenta and maternal environment, which mediates the change of insulin sensitivity. CaMK2*β* regulates a range of processes, including metabolism and insulin sensitivity [135]. The role of CaMK2 *β* in the pathophysiology of GDM needs to be further studied. Another study found that there were different protein expressions of spectrin alpha erythrocytic (SPTA)-1, CAMK2*β*, PAPP-A, perilipin 4, fatty acid binding protein (FABP) 4, and hexokinase-3 in peripheral blood of patients with GDM compared with normal controls. Interestingly, these proteins have previously been shown to be expressed differently in insulin resistance [131].

## 4. Conclusions and Prospect

In recent years, interest has been emerging in the research of exosomes during pregnancy. Exosomes play a crucial role in normal physiological pregnancy, from exosomal loading to maternal-fetal interface communication in pathological pregnancies such as GDM. A study found that visceral fat thickness in early pregnancy is more accurate in predicting GDM than BMI [136]. Another prospective study found that visceral fat thickness may predict GDM by regulating the miRNA-148 family of adipose-derived exosomes [137]. This suggests that the assessment of changes in exosomal content and composition may be the basis for the identification of potential diagnostic markers for GDM. In addition, further investigation of the mechanism of exosomes in normal pregnancy and GDM will increase our understanding of the function of circulating exosomes in patients with GDM and the pathophysiological mechanism of GDM, providing the basis for improving pregnancy outcome.

## Figures and Tables

**Table 1:** The upregulated expression levels of RNAs in GDM and the possible mechanism involved in GDM.

Sample	Upregulated	Related mechanism	Publication
Serum and urine	miR-429	IRS-1: a target gene of miR-429	[57]
Serum	miR-1323	Inhibit trophoblast cell viability via inhibiting TP53INP1	[58]
Serum	miR-16-5p, miR-29a-3p, and miR-134-5p	—	[59]
Placenta	miR-222	Suppress inflammatory response by promoting CXCR4 and inactivating NLRP3 inflammasomes	[60]
Serum	miR-2467	Adiponectin: a target gene of miR-2467	[61]
Placenta	miR-140-3p	Relate to defective placental IR signaling	[62]
Blood	miRNA-223	—	[63]
Serum	miR-195-5p	Inhibit cell viability and proliferation and promote apoptosis by targeting EZH2	[64]
Blood	miR-330-3p	INS-1 cell dysfunction	[65]
Peripheral blood	miR-770-5p	Influence pancreatic *β*-cell function	[66]
Placental macrophages	miR-657	Regulate macrophage proliferation, migration and polarization	[67]
Serum	miRNA-19a and miRNA-19b	—	[68]
Placenta-derived mononuclear macrophages	miR-657	Influence inflammatory response via IL-37/NF-*κ*B signaling axis	[69]
Plasma	miR-137	HG-induced VEC dysfunction	[70]
Whole blood cells	miRNA-340	PAIP1: a miRNA-340 target gene	[71]
Placenta tissue	miR-503	Regulate pancreatic *β*-cell function by targeting the mTOR pathway	[72]
Plasma	miR-16-5p, miR-17-5p, and miR-20a-5p	Correlate with IR	[35]
Placenta	miR-98	Link to the global DNA methylation by targeting Mecp2	[73]
HUVECs	circ_0074673	Regulate the proliferation, migration and angiogenesis of HG-HUVECs via the miR-1200/MEOX2 axis	[74]
Plasma	circ_0008285	circ_0008285 may maintain the HTR-8/SVneo trophoblast cell function by the PI3K/Akt signaling pathway	[45]
Placental tissues	Circ-PNPT1	Promoted HG-induced trophoblast cell biological dysfunction through miR-889-3p/PAK1 axis	[52]
Peripheral blood	ERMP1, TSPAN32, MRPL38, and RPL13P5	RPL13P5 involved in insulin resistance via the PI3K-AKT and insulin signaling pathways	[75]
Umbilical cord blood exosomes	Lnc-RXYLT1-3 : 2, lnc-TFDP2-7 : 2, lncCOX17-2 : 3, and lnc-ZBTB46-3 : 6	Most of the exosomal lncRNAs harbored miRNA binding sites	[16]
Plasma	MEG8	—	[76]
Placental tissues	MALAT1	Associate with inflammation and the proliferation, invasion, and migration of placental trophoblastic cells via modulating the TGF-*β*/NF-*κ*B signaling pathway	[77]
Blood and placental villous tissues	MEG3	miR-345-3p: a target; inhibit HTR-8/SVneo cell viability, and prevent cell migration and invasion in addition to inducing cell apoptosis	[78]
Serum	MALAT1	—	[79]
Plasma	SOX2OT	—	[80]

Abbreviations: IRS: insulin receptor substrate; CXCR4: C-X-C chemokine receptor type 4; IR: insulin resistance; EZH2: enhancer of zeste homolog 2; IL: interleukin; NF-*κ*B: nuclear factor *κ*B; HG: high glucose; VEC: vascular endothelial cell; mTOR: mammalian target of rapamycin; Mecp2: methyl CpG binding protein 2; HUVECs: human umbilical vein endothelial cells; PI3K: phosphatidylinositol 3-kinase; Akt: protein kinase B; PAK1: p21 activated kinase 1; TGF-*β*: tumor growth factor *β*; MEG3: maternally expressed gene 3.

**Table 2:** The downregulated expression levels of RNAs in GDM and the possible mechanism involved in GDM.

Sample	Downregulated	Related mechanism	Publication
Plasma	miR-574-5p and miR-3135b	Associate with insulin signaling pathway	[81]
Placenta tissues	miR-362-5p	Promote cell proliferation and inhibited apoptosis via targeting GSR and activating PI3K/Akt pathway	[82]
Placenta	miR-30d	Affect trophoblast cell functions by targeting RAB8A	[83]
Placenta and plasma	miR-96-5p	Increased the viability of trophoblasts	[84]
Placenta-derived macrophages	miR-6869-5p	Prevent from inflammation and inducing M2 macrophages	[85]
Placenta villous	miR-9 and miR-22	Alter placental glucose metabolism by targeting GLUT1 and HK2	[86]
Blood leukocytes	miR-4646	—	[87]
Placental tissue and peripheral blood	miR-345-3p	Inhibit HTR8-/SVneo cell apoptosis and promote cell proliferation and migration by targeting BAK1	[88]
Leukocytes	miR-155-5p	—	[89]
Peripheral blood	miR-193b	Inhibit autophagy and apoptosis by targeting IGFBP5	[90]
Serum	miR-29a/b	—	[91]
Serum and placenta	miR-21	Inhibit cell growth and infiltration by upregulating PPAR-*α*	[92]
Placenta	miR-29b	HIF3A: a direct target of miR-29b	[93]
Serum and placenta	miR-185	HOMA-IR ↓	[94]
Serum and placenta	miR-132	Enhance the trophoblast cell proliferation	[95]
Placental of rats	miRNA-221	Regulate proliferation, apoptosis and insulin secretion in islet *β* cells through targeting PAK1	[96]
Placenta tissues	miR-96	Regulate PAK1 expression, insulin secretion, and *β*-cell function	[97]
Blood	miR-494	Improve pancreatic *β*-cell dysfunction by targeting PTEN	[98]
Plasma	hsa_circRNA_102893	—	[99]
Plasma	circ_0001173	—	[45]
Placenta and plasma	hsa_circ_0005243	Induce trophoblast cell dysfunction and inflammation by the *β*-catenin and NF-*κ*B signal pathways	[100]
Placenta	circ_5824, circ_3636, and circ_0395	—	[101]
Plasma	SNHG17	—	[102]
Umbilical cord blood exosomes	Lnc-TBC1D30-4:1, ENST00000596839.1, lncZNF800-1 : 1, lnc-EIF4ENIF1-1 : 1, and lnc-ATP8B3-3 : 1	Most of the exosomal lncRNAs harbored miRNA binding sites	[16]
Placenta	PVT1	Disrupt the function of trophoblast cells through PI3K/Akt pathway	[103]
Blood leukocytes	Pax8-AS1	—	[87]

Abbreviations: GSR: glutathione-disulfide reductase; PI3K: phosphatidylinositol 3-kinase; Akt: protein kinase B; BAK1: BCL2-antagonist/killer 1; IGFBP5: insulin-like growth factor-binding protein 5; HOMA: homeostasis model assessment; IR: insulin resistance; PAK1: p21-activated kinase 1; PTEN: phosphatase and tensin homolog; NF-*κ*B: nuclear factor *κ*B; Pax8-AS1: paired box 8 antisense 1.
